# Beyond Visual Consensus: Tiered Reference Framework for AI Cystoscopy Studies

**DOI:** 10.2196/101910

**Published:** 2026-06-18

**Authors:** Ahmet Murat Bayraktar, Bilgi İşler

**Affiliations:** 1 Department of Urology, Konya City Hospital, Akabe Mah, Adana Çevre Yolu Cad. No.135 Karatay, Konya, 42020, Turkey, 90 332310000 ext 21022

**Keywords:** multimodal, large language model, AI, cystoscopy, diagnostic reasoning, finding description, biopsy indication, bladder tumor, artificial intelligence

We read with great interest the study by Shih et al [1], a valuable contribution to the emerging field of artificial intelligence (AI)–assisted cystoscopic diagnosis. Their blinded evaluation of four multimodal large language models across 401 images encompassing 40 cystoscopic finding subcategories provides important insights into current model capabilities. We wish to raise a methodological consideration regarding the reference standard that may inform the interpretation of the reported findings.

The reference standard in this study was established through visual consensus between two urologists, without histopathological confirmation. While interexpert agreement was satisfactory (κ=0.81), cystoscopic impression alone has well-documented limitations. Cina et al [2] demonstrated that experienced urologists could not reliably distinguish between low- and high-grade papillary lesions endoscopically, with complete grade-stage concordance with histopathology in only 70.3% of cases and a specificity of just 57% for predicting lamina propria invasion. A visually derived reference standard thus carries inherent diagnostic uncertainty.

This concern is particularly relevant for lesion categories central to the study’s 7-class task. Carcinoma in situ (CIS) is notoriously difficult to identify under white light cystoscopy; blue light cystoscopy studies have demonstrated that approximately one-third of CIS lesions are missed by white light alone [3]. Similarly, the frequent misclassification of papilloma as papillary urothelial carcinoma—acknowledged by the authors as reflecting substantial macroscopic overlap [1]—underscores that definitive classification of these entities requires histological evaluation of architectural and cytological features indistinguishable on endoscopic inspection.

The AI-assisted cystoscopic diagnosis literature has converged on histopathological confirmation as the reference standard. Foundational work such as CystoNet was trained and validated on histologically confirmed lesions [4], and a recent systematic review by Hengky et al [5] restricted inclusion to studies using histopathology as the reference standard. This consensus reflects the clinical reality that the categorical distinctions central to bladder lesion classification—low- versus high-grade carcinoma, CIS versus inflammation, papilloma versus carcinoma—ultimately rest on histological criteria and drive subsequent management.

We acknowledge the logistical challenges of obtaining histopathology for every image in a large, multisource dataset, particularly for benign-appearing or nonresected findings. We, therefore, suggest that future benchmarking studies adopt a tiered reference framework: (1) histopathologically confirmed labels for all lesions undergoing biopsy or resection, encompassing the full malignant spectrum; (2) enhanced cystoscopy correlation (blue light or narrow band imaging) as an intermediate standard, particularly for CIS [3]; and (3) consensus visual labels—explicitly flagged as lower confidence—for benign categories unlikely to undergo biopsy in routine practice. Stratified performance reporting under such a framework would allow readers to separate genuine algorithmic limitations from ambiguity inherent to the reference standard, providing a more clinically meaningful evaluation.

## Abbreviations

AI: artificial intelligence
CIS: carcinoma in situ
